# Aspirin, lysine, mifepristone and doxycycline combined can effectively and safely prevent and treat cancer metastasis: prevent seeds from gemmating on soil

**DOI:** 10.18632/oncotarget.6038

**Published:** 2015-10-08

**Authors:** Liyuan Wan, Haiyan Dong, Huo Xu, Ji Ma, Yewei Zhu, Yusheng Lu, Jichuang Wang, Ting Zhang, Tao Li, Jingjing Xie, Bo Xu, Fangwei Xie, Yu Gao, Jingwei Shao, Xiaohuang Tu, Lee Jia

**Affiliations:** ^1^ Cancer Metastasis Alert and Prevention Center, and Pharmaceutical Photocatalysis of State Key Laboratory of Photocatalysis on Energy and Environment, College of Chemistry, Fuzhou University, Fuzhou, China; ^2^ Obstetrics and Gynecology, Fuzhou General Hospital, Fuzhou, China; ^3^ Department of Medicine Oncology, East Hospital of Xiamen University, Fuzhou, China; ^4^ Department of Surgery, East Hospital of Xiamen University, Fuzhou, China

**Keywords:** cancer metastasis, cell adhesion molecules, drug combination, metastasis chemoprevention, HAMPT

## Abstract

Recent scientific advances have increased our understanding of the cancer metastatic complexities and provided further impetus for new combination therapies to prevent cancer metastasis. Here, we demonstrated that a combination (HAMPT) of aspirin, lysine, mifepristone and doxycycline can effectively and safely prevent cancer metastasis. The pharmaceutically-formulated HAMPT inhibited adhesion of cancer cells to either endothelial cells or extracellular matrix via down-regulating cell adhesion molecules ICAM-1 and α_4_-integrin. HAMPT inhibited the cloak effect by activated platelets on cancer cells, thereby interfering adhesion and invasion of cancer cells to the underlying stroma. At the effective concentration, HAMPT induced cancer cells into dormancy with minor inhibition on cell viability. Four-day pretreatment followed by 30-day oral administration of HAMPT (33.5-134 mg/kg) to the mice inoculated with cancer cells produced significant inhibition on cancer metastasis dose-dependently without marked side effects. Fifty-day rat toxicity study with HAMPT at doses (335-1340 mg/kg) 20-fold higher than its therapeutic dose produced no significant toxicity. Interestingly, the acute toxic death could not be reached at the maximum administrable dose (5 g/kg). This proof-of-concept study provides the first conceptual evidence that cancer metastasis can be controlled by using affordable old drugs to restrain circulating tumor cells from gemmating on the metastatic soil without the need for cytotoxicity.

## INTRODUCTION

The increased number of cancer survivors is the cause for celebration [1], but this expanding population has highlighted the problem of cancer relapse and metastasis after surgical removal of the primary tumors [2]. Indeed, the emergence of disseminated metastases remains the primary cause of mortality in cancer patients [3], which is the daily threat to the cancer survivors, among them 30-70% will eventually face the metastatic nightmares within 2-5 years after surgery.

The root cause of cancer metastasis can be traced down to the presence of circulating tumor cells (CTCs) or tumor-specific DNA in the blood of cancer survivors [4]. CTCs in cancer survivors often show a low rate of proliferation when cancer survivors are in remission and/or asymptomatic [5-6]. Thus current post-metastatic chemotherapeutic agents that are originally designed to target highly proliferating cancer cells could also be destructive to proliferating non-cancer cells, resulting in intolerable side effects. Hence, chemotherapeutics cannot be used in the asymptomatic cancer survivors [7]. When CTCs become activated, however, treatments directed against metastasis are too late because CTCs have already spread and seeded to various vulnerable tissues [8-9]. At that moment it is nearly impossible to stop or reverse the devastating cascade of cancer metastasis by using chemotherapeutics, partially due to the acquired or inherent resistance of the CTCs to the chemotherapeutics [10]. As a matter of fact, anticancer chemotherapy sometimes enhances metastasis formation [11]. Worse off, the activated CTCs possess more heterogeneity and drug tolerance than the normal cells [12].

Following logical dissection on the cancer metastasis pathways using tools of systems biology and systems pharmacology [13], we depict that primary tumor cells separate from neighboring epithelial cell-cell contacts and become the CTCs. They travel in a unidirectional path in the circulation, reside in the bone marrow and lymph node, and ultimately colonize distant organs to form the pre-metastatic niche or repopulate the primary site through self-seeding [9]. Many local microenvironment factors collectively decide whether and where CTCs colonize. These factors [14] include tumor-derived or locally-produced inflammatory factors [chemoattractants, transforming growth factor-β (TGF-β)], the diameter of capillary vessels, activated platelets that escort CTCs from immune cell recognition and attack, fibroblasts that upregulate fibronectin to establish a docking site for CTCs, immunosuppressive cell types such as Myeloid-derived suppressor cells (MDSCs) and natural killer cells (NK) that populate premetastatic niches permissive to CTCs colonization, acidification and adenosine triphosphate (ATP) production, activities of cell adhesion molecules, integrins, cyclooxygenase 2 (COX-2) and matrix metalloproteinase (MMP). On the other hand, if CTCs fail to adhere to the vascular endothelium, they may die through the blood shear stress, or a process termed anoikis, one form of detachment-induced apoptotic program that has been characterized in suspension cell cultures [15]. We proposed that the initiation of CTC adhesion to the local vascular endothelium is the first and important step for CTCs to start the metastatic cascade. Inhibition of the initial step may thus prevent consequential formation of the metastasis foci [16]. We then demonstrated that metapristone (the active mifepristone metabolite) has a safe and effective profile as a cancer metastasis chemopreventive agent by inhibiting adhesion of CTCs to vascular endothelium [17]. Very recently, we further demonstrated that both the extracts from the medicinal plant *Murraya exotica* and the engineered dual antibody coated nanomaterials have the similar safe and effective profile of inhibiting adhesion of CTCs to vascular endothelium [18-19].

Based on the above understanding of cancer metastatic processes, we designed, accordingly, a quadruple combination drug HAMPT (standing for highly active metastasis prevention therapy), which is consisted of mifepristone (RU486), aspirin, lysine and doxycycline. Each component of HAMPT targets the above-mentioned local microenvironment factors to comprehensively and synergistically interfere with cancer metastasis pathways. Here, we report pharmaceutical preparation and analysis of the HAMPT combination, its *in vitro* effects on cell viability and cycle distribution, adhesion between cancer and endothelial cells, cloak effect by the activated platelets on cancer cells, and expression of adhesion molecules. We also conducted the *in vivo* experiments to analyze synergistic effect of HAMPT on the well-established Humphries's metastasis animal model [20], and examined the long-term safety profile of HAMPT.

## RESULTS

### No physicochemical interaction among the ingredients

**Figure 1:**
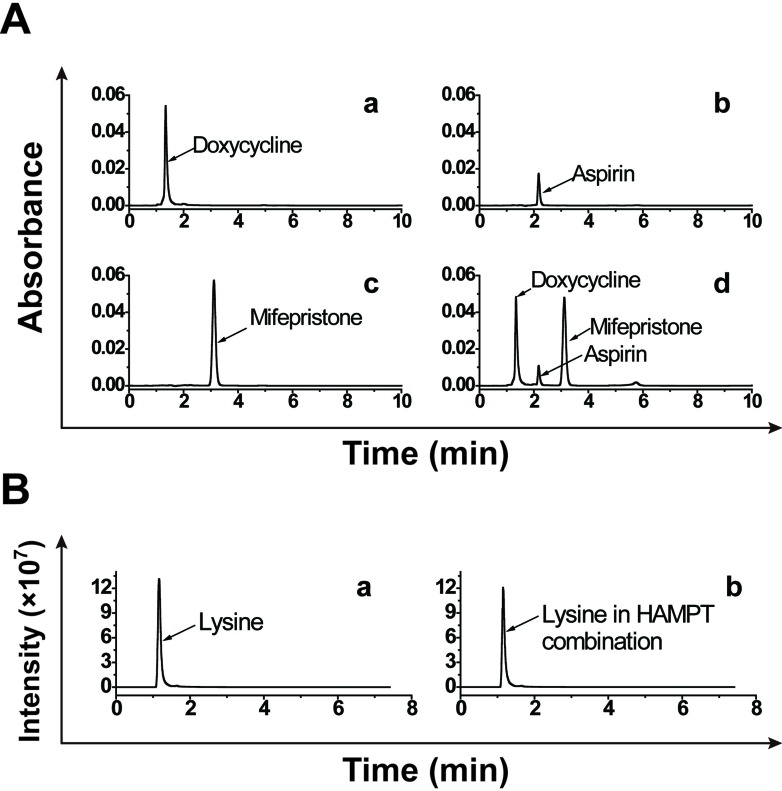
No significant physicochemical drug-drug interaction among the HAMPT ingredients The ingredients were dissolved in methanol-water solvent (5:1; v/v) at the final concentration of 10 μg/mL alone (**A.**-a, b, c and **B.**-a) or in the mixture (**A.**-d and **B.**-b) for 24 h at room temperature. Chromatogram, peak areas and retention times of individual ingredients and their mixture were determined by the HPLC method (for mifepristone, aspirin and doxycycline hyclate) and UPLC-MS/MS (for lysine).

To rule out physicochemical interactions among the individual active ingredients in HAMPT, we examined differences in chromatogram and peak areas of each ingredient alone, or in the HAMPT mixture at room temperature incubated in the same solvent for 24 h. The final concentration of each ingredient alone and in the mixture was kept same. As shown in Figure 1, each ingredient, whether it is in a single one or mixed with other drugs, exhibited the similar chromatogram, peak areas and retention times under the same chromatographic conditions as well as mass ionization and spectrometric conditions. The result indicated no significant physicochemical drug-drug interaction among these ingredients after they were mixed in the solvent under the preparation condition.

### HAMPT primarily inhibits CTC adhesion with minor effect on cell viability

**Figure 2:**
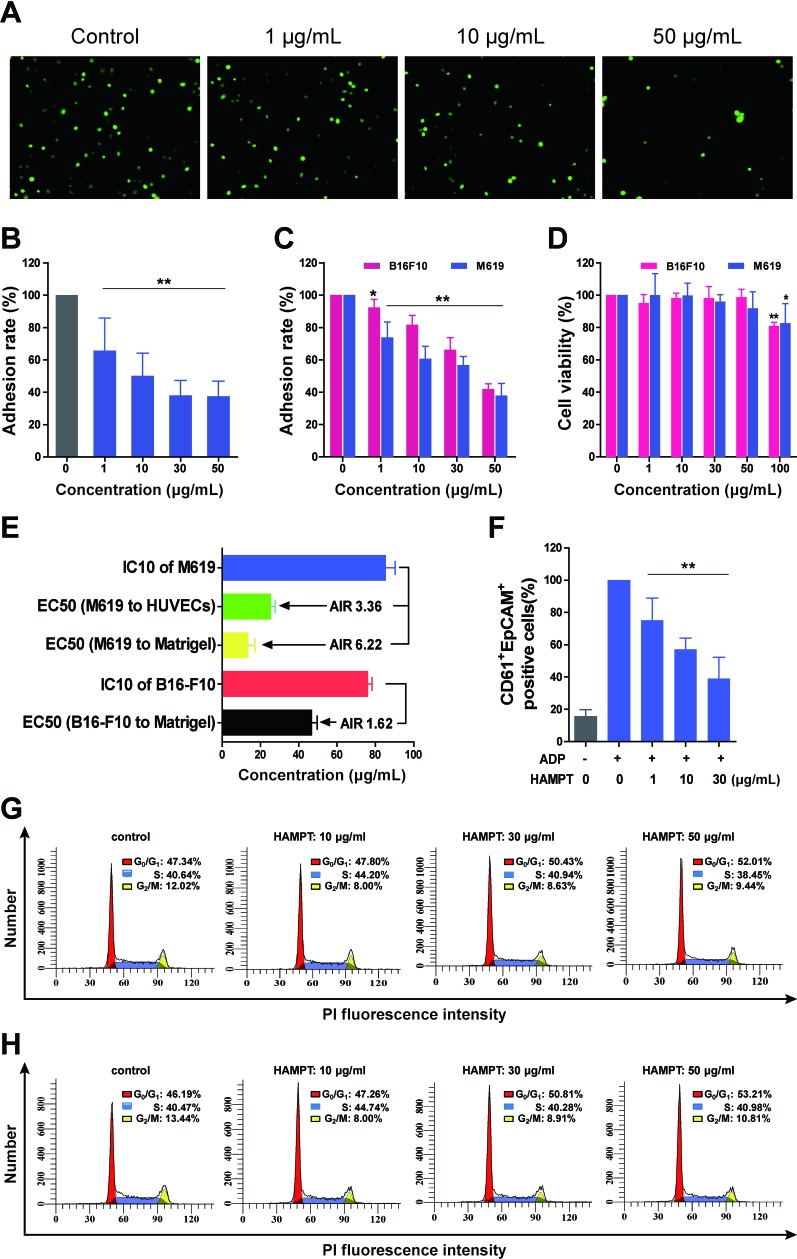
Cellular and molecular mechanisms of actions of HAMPT by which it exerts cancer metastasis chemoprevention **A.** Representative microscopic observation of the inhibition by HAMPT at 0, 1, 10, and 50 μg/mL on adhesion of M619-GFP to HUVECs; The quality of HUVECs was checked by our method (16). **B.** Concentration–dependent effect of HAMPT on adhesion of human melanoma cells M619 to HUVECs. **C.** Concentration–dependent effect of HAMPT on adhesion of M619 and B16-F10 cells to Matrigel. **D.** HAMPT did not show significant effect on viability of human melanoma M619 and mouse melanoma B16-F10 cells until it reached 100 μg/mL. **E.** Adhesion inhibition ratio (AIR) of HAMPT defined by dividing growth inhibition IC_10_ by adhesion inhibition EC_50_. HAMPT showed a good AIR at inhibiting adhesion of human M619 to HUVECs, suggesting that HAMPT functions primarily by inhibiting cell-cell adhesion, rather than cell killing. **F.** HAMPT, in a concentration-dependent manner, prevents cancer M619 cells from cloak or aggregation by activated human platelets. Effect of HAMPT on % distribution of M619 **G.** and B16-F10 (H) cells in different phases of the cell cycle measured by using flow cytometry; the result indicates that HAMPT treatment increases cell population in the G_0_/G_1_ phase, while reducing cell population in S and G_2_/M phases. The quantitative analysis represents the means±SD of three independent experiments as compared with the untreated control (*P <0.05, **P < 0.01).

The adhesion and invasion of CTCs to endothelial cells are considered as the important initial step of distant cancer metastasis. We first examined the effect of HAMPT on cell hetero-adhesion between human endothelial cells and cancer cells M619. After co-incubation of M619 with HUVECs, the fluorescent quantification (Figure 2A) revealed that HAMPT produced significant inhibition on the adhesion in a dose-dependent manner (Figure 2A, 2B). For example, at 50 μg/mL, HAMPT inhibited adhesion of M619 to HUVECs activated by interleukin-1 beta (IL-1β) by 37.3 ± 9.5 % in comparison with the control. IL-1β is commonly used in the static adhesion assay in this laboratory because it enhances expression of adhesion proteins vascular cell adhesion molecule-1 (VCAM-1) and intercellular adhesion molecule-1 (ICAM-1) on endothelium [16].

We then assessed the effect of HAMPT on adhesion of cancer cells B16-F10 and M619 to Matrigel that was used as the artificial extracellular membrane (ECM) [17]. MTT assay revealed that HAMPT inhibited adhesion of both human and mouse melanoma cancer cells to Matrigel in a dose-dependent manner (Figure 2C). For instance, the adhesion of M619 and B16-F10 cells to Matrigel was inhibited by 41.8 ± 3.4 and 37.7 ± 7.6 %, respectively, in the presence of 50 μg/mL HAMPT (*P* < 0.01).

The effect of HAMPT on cell viability was examined on mouse melanoma B16-F10 and human melanoma M619 cell lines, respectively. HAMPT at doses of 1, 10, 30, and 50 μg/mL did not produce statistically significant changes in viability of the two cell lines. However, when HAMPT concentration was increased to 100 μg/mL, a statistically significant inhibition on the growth of these cell lines was observed in comparison with the untreated controls (Figure 2D).

To quantitatively characterize a cancer metastasis chemopreventive that is different from cytotoxic anticancer drugs in the mechanism by which a cancer metastasis chemopreventive inhibits adhesion of cancer cells to endothelial cells, instead of killing cells, we herein created the adhesion inhibition ratio (AIR) to define this type of drugs. The ratio is calculated by dividing IC_10_ (the mean drug concentration causing a 10% growth inhibition of the cells) by EC_50_ (the mean drug concentration causing a 50% inhibition of the hetero-adhesion between cancer cells and endothelial cells). The larger the AIR is, the more likely the drug works as a cancer metastasis chemopreventive by inhibiting the hetero-adhesion, rather than as a cytotoxic drug that inhibits the cell hetero-adhesion by cell killing and may have side effects. As shown in Figure 2E, the AIR value of HAMPT in inhibiting growth of M619 and its adhesion to HUVECs is 3.36. The AIR values of HAMPT in inhibiting growth of M619 and B16-F10 and their corresponding adhesion to Matrigel are 6.22 and 1.62, respectively. The result indicates that the cellular mechanism of HAMPT is by, primarily, directly inhibiting hetero-adhesion between cells, rather than killing cells.

### HAMPT prevents cancer cells from cloaked or aggregated by platelets

The flow cytometry analysis showed that M619 was EpCAM^+^CD61^−^, whereas, platelets were EpCAM^−^CD61^+^. Therefore, the platelet-cloaked cancer cell aggregates should be EpCAM^+^CD61^+^. When the platelets were at rest, the EpCAM^+^CD61^+^ platelet-cloaked M619 aggregates were rarely to be detected. However, once activated by ADP after 10 min incubation, the platelet-cloaked M619 aggregates were significantly increased. HAMPT caused a significant concentration-dependent decrease in platelet-cloaked M619 aggregates by 75.2± 13.7 (1 μg/mL), 57.3± 6.9 (10 μg/mL) and 39.1± 13.2% (30 μg/mL), respectively, compared to the ADP-activated platelets in the absence of HAMPT (Figure 2F). The present study indicated that HAMPT inhibited platelet-cloaked M619 aggregation induced by ADP. It is well-demonstrated that platelets contribute to tumor metastasis [21].

### Effect of HAMPT on cell cycle distribution

Cell growth in tumor is carefully controlled by regulating the cell cycle. To further determine how HAMPT affect the viability of M619 and B16-F10 cells, cell cycle distribution was analyzed by flow cytometry to reveal the percentage of the cells arrested in the phases of G_0_/G_1_, S, and G_2_/M, respectively, after HAMPT treatment. The results indicated that HAMPT produced a concentration-dependent increase in M619 (Figure 2G) and B16-F10 (Figure 2H) cell population in G_0_/G_1_ phase, and decreases in the cell population in S and G_2_/M phases, suggesting that HAMPT could drive cells into a resting phase (G_0_) where the cells have left the cycle and stop dividing, or into G_1_ phase where they are ready for DNA synthesis, while decrease cell population in DNA synthesis and mitosis.

### Inhibition by HAMPT on expression of cell adhesion molecules

**Figure 3:**
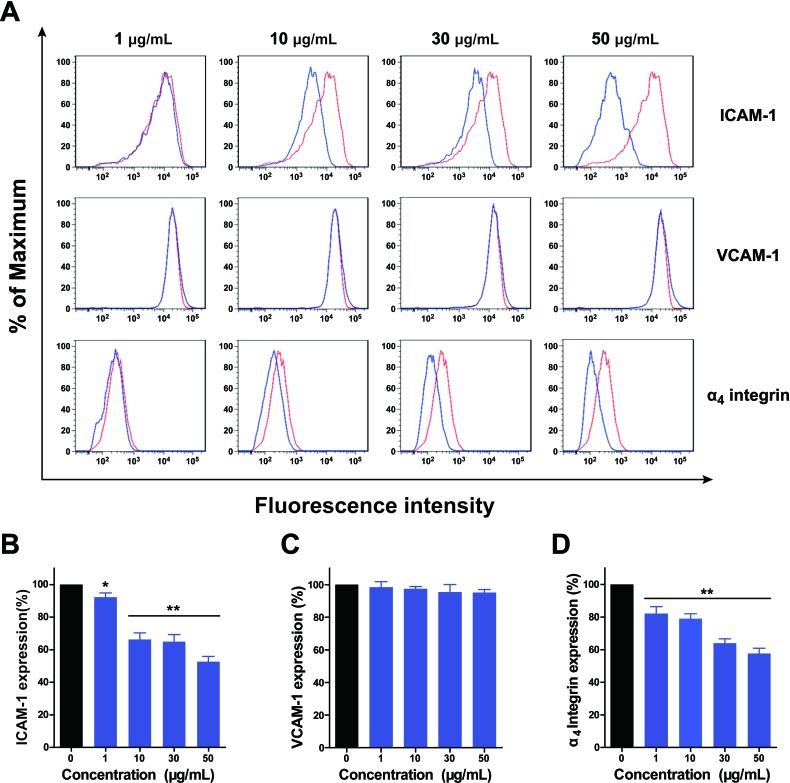
Flow cytometry analysis of the effect of HAMPT on expression of CAMs by HUVECs and expression of ɑ_4_ integrin by melanoma B16-F10 cells **A.** HAMPT showed inhibitory effect on ICAM-1 expression in a dose-dependent manner. The red and blue lines represent ICAM-1 expression by HUVECs before and after HAMPT treatment, respectively. **B.** and **C.** show, respectively, the percentage of ICAM-1 and VCAM-1 expression in the presence and absence of HAMPT. **D.** shows that the expression of ɑ_4_ integrin by melanoma B16-F10 cells was down-regulated by HAMPT. Results (*n* = 3 per group) are expressed as the percentage of the untreated samples (*p < 0.05, **p < 0.01).

Since HAMPT was defined as a cancer metastasis chemopreventive combination that showed high AIR and specifically inhibited the cell hetero-adhesion, we explored if HAMPT could affect the expression of those cell adhesion molecules by using flow cytometry as we described previously [16-17]. In Figure 3A, the FACS histogram of a typical experiment showed the effect of HAMPT on IL-1β-induced ICAM-1 expression illustrated by fluorescence intensity on the X-axis and % of maximum on the Y-axis. The HUVECs were pretreated with 1 ng/mL IL-1β for 4 h, followed by treatment with HAMPT for another 24 h. HAMPT down-regulated ICAM-1 expression in a dose-dependent manner (Figure 3A, 3B) by 63.3 ± 4.0 % (10 μg/mL) and 52.7 ± 3.2 % (50 μg/mL). Whereas, HAMPT (1, 10, 30 and 50 μg/mL) did not show significant dose-dependent down-regulation of VCAM-1 expression (Figure 3C). Flow cytometric analysis revealed that ɑ_4_ integrin is an adhesion ligand expressed on the surface of B16-F10 cells and HAMPT caused a significant decrease in ɑ_4_ integrin expression by 57.7 ± 3.2 % at 50 μg/mL in the B16-F10 cells (Figure 3D).

### Analyses of different combinations and the related additive and synergistic effects

**Figure 4:**
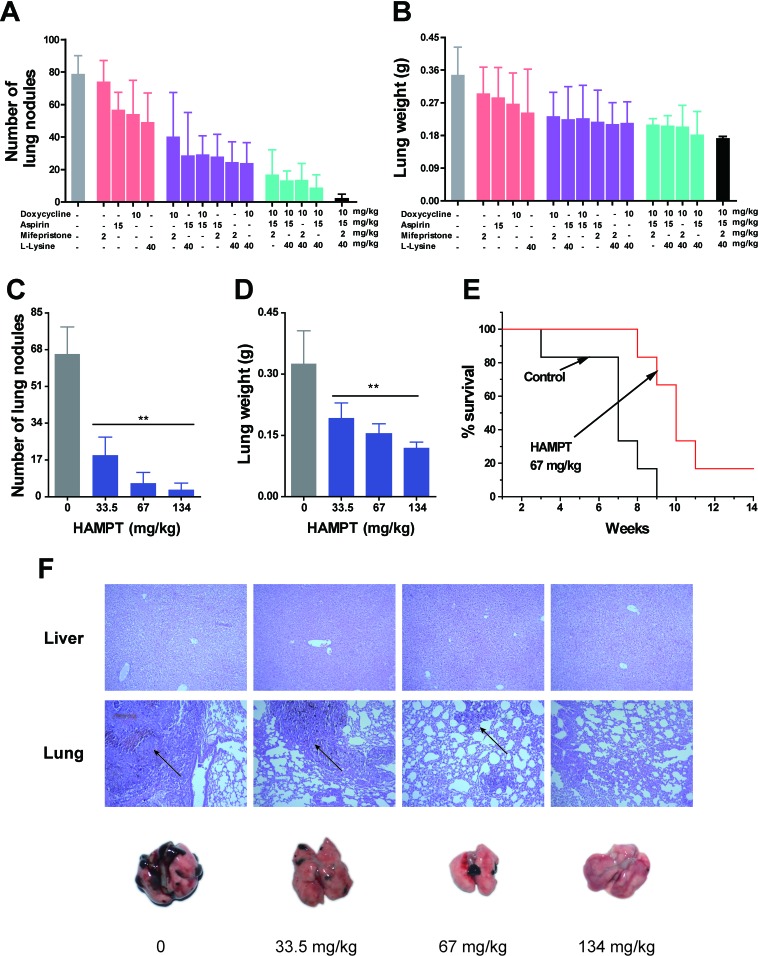
Effects of HAMPT on melanoma experimental mouse model Female C57BL/6 mice were pretreated with oral HAMPT for four days at three dose levels, and the mice were intravenously injected with 3×10^4^ B16-F10 melanoma cells via the tail vein followed by oral HAMPT treatment for 30 days. **A.** and **B.**, effects of various combinations among doxycycline, aspirin, mifepristone, and L-lysine at medium dose of HAMPT on lung nodules and lung weight; the results showed that HAMPT (the far right bar) was the best combination. **C.** and **D.**, dose-dependent inhibition of HAMPT on lung metastasis nodules and overall lung metastatic weight. **E.** Kaplan-Meier survival analysis showed cumulative post-inoculation survival rates of the mice administrated with the median dose of HAMPT in comparison with the untreated control. **F.** Representative lungs from an experiment to show the number of surface melanoma colonies after the 30-day treatment with low, medium, and high doses of HAMPT in comparison with the control. The low panel shows H&E staining of paraffin embedded lungs of C57BL/6 mice treated with the corresponding HAMPT, and the arrows indicate the metastatic area. The result indicated HAMPT is a safe and effective metastasis preventive drug.

The metastasis-preventing activity of HAMPT was tested using previously characterized experimental metastatic murine model [20]. Briefly, B16-F10 murine melanoma cells (3×10^4^/mice), a line for high colonization *in vivo*, were injected into the lateral tail vein of C57BL/6 mice. To optimize the combination strategies for the most potent inhibition on the lung metastasis, mifepristone, aspirin, lysine and doxycycline hyclate were orally administered to the lung metastatic mouse model alone, or in dual or triple combination for 30 days, and the metastatic inhibition effects of different combinations were evaluated based on the medium dose of HAMPT (67 mg/kg/day). As shown in Figure 4A and 4B, HAMPT exhibited the most potent inhibition on the lung metastasis (the far right bar), followed by triple combinations (the green bars), and then dual combinations (the purple bars). The result confirmed the pharmacological necessity of the four drug combination to comprehensively inhibit cancer metastasis. We then used the following equation to determine if the individual combination was additive or synergistic based on the *in vivo* experimental therapy data: q = E_A+B_/E_A_+E_B_-E_A_×E_B_ [22], where, E_A+B_, E_A_, and E_B_ denote the average inhibitory effects of any drug A and B combined, or alone, on the number of lung tumor nodules and lung weight. When the q is ≤0.85, the result suggests an antagonism between the combination; when the q is 0.85 < q < 1.15, an additive effect; and q≥1.15, a synergistic effect between the combination. As analyzed and shown in Table S1, there was no antagonism between different combinations, and many combinations can be categorized as “additive”, including HAMPT, and some, as “synergistic”.

### Inhibition of *in vivo* cancer metastasis by HAMPT treatment

We then determined the optimal dose for the HAMPT combination based on optimal metastasis-preventing benefit, i.e., affordability, maximal efficacy and a minimal toxicity [23], and re-appraised the doses of those agents that have already become the standard of care according to animal-human equivalent doses converted from body surface area ratio [24]. Three doses of each individual drug were chosen to cover wider therapeutic window and explore dose-related efficacy and toxicity.

Thirty-day treatment with HAMPT diminished the lung metastasis in a dose-dependent manner (Figure 4). Confocal imaging after staining for vascular endothelium and B16-F10 cells showed that vehicle-treated mice harbored large lung metastatic lesions that had efficiently extravasated, whereas mice treated with HAMPT exhibited a strong inhibition towards smaller micrometastases, resulting in a significant decrease in both lung tumor nodules (Figure 4C) and lung weight (Figure 4D). Notably, the lung weight of the mice treated with high dose of HAMPT was kept within the normal range. Microscopic quantification of multiple lung sections demonstrated statistically significant inhibitory effects of HAMPT on the overall lung metastatic burden quantified by the overall lung weight (Figure 4D) as well as on the size distribution of the lung metastasis lesions quantified by the overall lung nodules (Figure 4C). The vehicle-treated control mice had massive growth of tumor, which was significantly reduced to countable colonies in a HAMPT dose-dependent manner. The Kaplan-Meier estimate showed that the mean survival rates *versus* time of HAMPT-treated mice were significantly higher than those of the untreated control (Figure 4E). Histopathological H&E staining of various lung sections showed that HAMPT produced significant dose-dependent decrease in B16-F10-induced metastatic lesion and tissue density in the treated mice in comparison with the untreated controls (Figure 4F). Upper panel (Figure 4F) of histopathological H&E staining of various liver sections showed that HAMPT was safe after a long period of oral administration.

### Acute and subacute toxicity studies with HAMPT

The studies were conducted as we described previously [25]. Briefly, HAMPT (2, 3 and 5 g/kg) was administered by gavage to groups of mice (10 males, 10 females per group), while the control group received the vehicle only. The unusual behaviors, adverse effects and toxic signs occurred after administration of HAMPT were monitored continuously for 1 h and then intermittently for 4 h after the oral administration. The mice were closely observed over a period of 24 h and for a period of 14 days. No deaths or hazardous signs were observed during the 14-day study.

**Figure 5:**
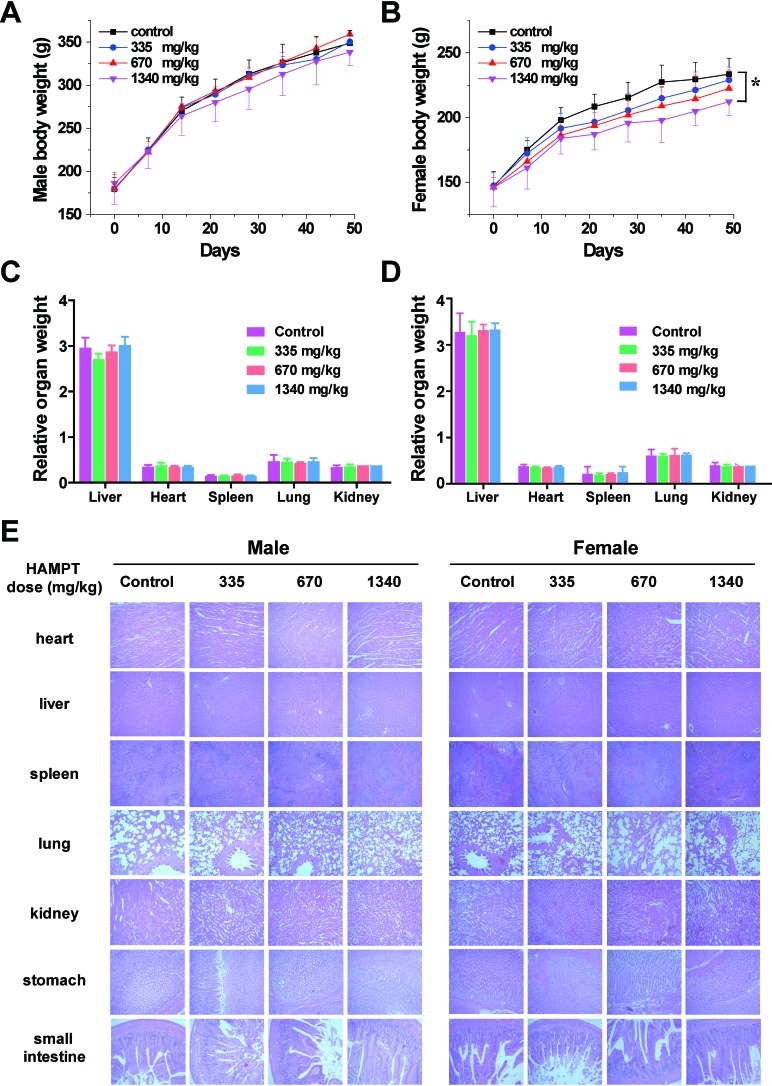
Safety profile of HAMPT administrated at high doses for consecutive 50 days Effect of 50-day HAMPT on body weight of male **A.** and female **B.** rats, and their main organ weights (**C.**, male; **D.**, female); **E.** histopathological analysis of the main organs of the control and HAMPT-treated rats. Photomicrographs (scale ×10) of the sections of the main organs showed no significant histopathological changes in the HAMPT-treated groups, especially in the highest dose group (1340 mg/kg).

For the subacute toxicity study, the Sprague-Dawley rats (5 males, 5 females per group) were administered with oral HAMPT (335, 670 and 1340 mg/kg/day) for 50 days, and their body weights were recorded weekly (Figure 5A, 5B) No toxic signs (such as piloerection, unusual locomotor activity) were observed during the subacute toxicity study. The rats were sacrificed on the 51st day, the blood samples were collected from common carotid for blood chemistry analysis (Table 1), and major tissues were freshly harvested and fixed in 10% buffered formaldehyde solution (Figure 5C, 5D, 5E). Following the histopathological processes, the tissues were stained with H&E and examined microscopically. In general, the body weight gain of the treated rats was normal in comparison with the control group except that the female rats receiving 1340 mg/kg/day of HAMPT showed significantly slow gain in body weights (212.2 ± 10.7 *versus* 233.5 ± 12.3 g of the control, *P* < 0.05; Figure 5B) as well as the increase in activity of liver alanine aminotransferase (70.8 ± 26.0 *versus* 43.7 ± 6.7 U/L of the control; *P* < 0.05) and direct bilirubin (0.4 ± 0.3 *versus* 0.8 ± 0.2 of the control; *P* < 0.05). With the exception of the latter two changes, no other significant changes in female blood chemistry (Table 1) were observed from all groups. Interestingly, both the body weight (Figure 5A) and blood chemistry (Table 1) of male rats showed no significant change compared with control group.

**Table 1:** Effects of oral HAMPT on blood chemistry of SD rats treated for 50 consecutive days

		Male				Female			
Item	Unit	Control	HAMPT (335 mg/kg)	HAMPT (670 mg/kg)	HAMPT (1340 mg/kg)	Control	HAMPT (335 mg/kg)	HAMPT (670 mg/kg)	HAMPT (1340 mg/kg)
WBC	10^9^/L	7.8 ± 0.2	7.8 ± 0.8	8.1 ± 1.0	8.8 ± 0.7	7.8 ± 2.9	8.8 ± 1.0	8.1 ± 0.6	8.6 ± 0.7
NEUT	%	15.4 ± 2.4	19.6 ± 5.8	17.1 ± 6.5	17.5 ± 9.1	12.9 ± 2.5	15.0 ± 3.3	11.6 ± 1.2	23.1 ± 5.7
LYMPH	%	81.4 ± 2.5	78.2 ± 6.2	76.1 ± 5.7	76.1 ± 13.3	82.8 ± 2.5	73.8 ± 6.8	83.7 ± 1.6	79.3 ± 5.7
EOS	%	1.4 ± 0.9	0.9 ± 0.2	1.1 ± 0.5	1.1 ± 0.4	0.9 ± 0.2	1.4 ± 0.9	1.4 ± 0.1	1.2 ± 0.8
BASO	%	0.1 ± 0.1	0.1 ± 0	0.1 ± 0.1	0.1 ± 0.1	0.1 ± 0.1	0.1 ± 0	0.1 ± 0	0.1 ± 0.1
RBC	10^12^/L	9.0 ± 0.6	8.8 ± 0.3	8.8 ± 0.3	8.7 ± 0.4	8.1 ± 0.1	7.8 ± 0.5	8.3 ± 0.3	7.5 ± 1.0
HGB	g/L	163.3 ± 4.0	160.5 ± 6.8	164.0 ± 3.6	165.3 ± 3.7	148.5 ± 3.5	150.0 ± 3.0	152.5 ± 9.1	153.0 ± 3.2
MCV	fL	54.6 ± 2.2	54.9 ± 1.3	56.1 ± 1.0	56.1 ± 1.4	56.2 ± 1.2	59.1 ± 4.1	56.5 ± 1.0	59.8 ± 2.1
MCH	Pg	18.3 ± 0.7	18.2 ± 0.4	18.7 ± 0.4	18.7 ± 0.1	18.4 ± 0.5	19.5 ± 1.2	18.4 ± 0.4	19.3 ± 0.3
MCHC	g/L	334.5 ± 4.0	331.3 ± 4.5	333.7 ± 1.3	334.7 ± 7.6	328.0 ± 2.8	325.5 ± 7.6	326.0 ± 1.8	324.5 ± 8.7
RDW	%	12.6 ± 0.6	12.6 ± 0.3	12.7 ± 0.7	12.7 ± 0.5	11.5 ± 0.4	17.8 ± 7.1	11.6 ± 0.3	13.0 ± 2.3
MPV	fL	8.0 ± 0.2	8.0 ± 0.2	7.9 ± 0.1	8.1 ± 0.2	7.9 ± 0.1	7.5 ± 0.3	7.8 ± 0.2	7.8 ± 0.2
PDW	%	8.8 ± 0.5	8.8 ± 0.5	8.7 ± 0.3	9.0 ± 0.2	8.5 ± 0.3	8.3 ± 0.3	8.2 ± 0.4	8.2 ± 0.2
Urea	mmol/L	5.3 ± 0.3	5.5 ± 0.4	4.9 ± 0.4	5.7 ± 0.8	6.8 ± 0.6	6.4 ± 0.6	6.2 ± 0.1	6.4 ± 0.6
Creatinine	μmol/L	40.5 ± 5.3	41.0 ± 2.8	42.7 ± 5.7	43.7 ± 6.7	42.7 ± 4.7	40.0 ± 5.7	39.5 ± 4.6	44.0 ± 7.8
TP	g/L	64.0 ± 2.2	62.3 ± 1.5	61.0 ± 2.5	61.7 ± 1.0	61.5 ± 3.4	65.2 ± 2.5	62.0 ± 2.2	64.3 ± 7.0
ALT	U/L	54.0 ± 9.7	52.3 ± 6.7	45.0 ± 1.6	53.7 ± 6.4	43.7 ± 6.7	50.7 ± 3.2	53.0 ± 4.6	70.8 ± 26.0*
AST	U/L	80.0 ± 6.3	87.3 ± 5.2	78.0 ± 8.9	81.2 ± 5.7	81.3 ± 8.1	77.0 ± 8.8	78.0 ± 8.9	103.0 ± 32.8
ALP	U/L	223.3 ± 29.3	217.8 ± 20.9	196.7 ± 3.5	192.2 ± 11.2	146.3 ± 16.2	135.2 ± 30.0	139.3 ± 21.7	134.5 ± 29.1
GGT	U/L	1.3 ± 0.5	1.5 ± 0.6	1.2 ± 0.5	1.0 ± 0	1.0 ± 0	1.0 ± 0	1.0 ± 0	1.0 ± 0
Tbil	μmol/L	2.1 ± 1.0	1.6 ± 1.2	1.8 ± 1.0	2.9 ± 1.6	2.1 ± 0.6	2.4 ± 1.0	2.4 ± 1.0	2.0 ± 0.5
DBil	μmol/L	0.6 ± 0.3	0.4 ± 0.1	0.5 ± 0.1	0.6 ± 0.1	0.8 ± 0.2	0.7 ± 0.3	0.7 ± 0.1	0.4 ± 0.3*

^a^Values represent the mean ± SD (n=5/group).

^b^WBC, white blood count; NEUT, neutrophil count; LYMPH, lymphocytes; EOS, eosinophils; BASO, basophil; RBC, erythrocyte count; HGB, hemoglobin concentration; MCV, corpuscular volume; MCH, corpuscular hemoglobin; MCHC, corpuscular hemoglobin concentration; RDW, red cell distribution width; PLT, platelets; MPV, platelet volume; PDW, platelet distribution width; TP, Total Protein; ALT, alanine aminotransferase; AST, aspartate aminotransferase; ALP, alkaline phosphatase; GGT, gamma glutamyl transpeptidase; Tbil, total bilirubin; DBil, direct bilirubin

At the end of the subacute experiment, representative organs were removed, weighed and compared with the body weight of the rats [26]. The relative weight (% of body weight, *n* = 5) of the rat kidneys, spleen, liver, heart and lungs in the HAMPT group and control group were shown in Figure 5C and 5D. There were no significant differences in the relative organ weight between the HAMPT-treated group and the control group. Figure 5E exhibits photomicrographs of the heart, liver, spleen, lung, kidney, stomach and small intestine. Histological features of the control and HAMPT-treated group rats showed normal structures.

## DISCUSSION

There is a compelling rationale for use of a combination of drugs with different mechanisms of action to target both a primary and compensatory pathway for cancer metastasis chemoprevention to minimize toxicity and maximize efficacy. In the present study, we demonstrated that HAMPT could be formulated in a novel quadruple drug combination without obvious physiochemical drug-drug interaction. The combination prevented and inhibited cancer metastasis in the well-validated animal model in a dose-dependent manner (Figure 4) through its inhibition on cell hetero-adhesion between cancer and endothelia cells (Figure 2) The inhibition included down regulation by HAMPT of ICAM-1 and integrin expression (Figure 3), the cloak effect (Figure 2F) caused by activated platelets to protect CTCs from immune attack and facilitate CTCs adhesion to endothelial cells for their subsequent gemmating and invasion to the underlying stroma. HAMPT also shows its ability to induce CTCs into cellular dormancy *via* a G_0_/G_1_ arrest or differentiation (Figure 2G) [27], and minor inhibition on cell viability. Thirty-day treatment of the mice with HAMPT (33.5, 67, 134 mg/kg/day) did not produce any significant drug-related organ toxicity, which was further confirmed by the 50-day subacute (5 g/kg) and the acute toxicity (335, 670 and 1340 mg/kg/day) studies. These data strongly suggest that HAMPT is a good cancer metastasis chemopreventive that could comprehensively and synergistically interfere with metastasis pathways while possessing a good safety profile.

The majority of the currently developed and marketed anticancer drugs are originally aimed at manipulating the primary tumor. Hardly any cancer therapies used at the moment interfere only with metastasis and there is an urgent need for drugs acting specifically on the metastatic processes, curing the minimal residual disease and being much less toxic than the current drugs [28]. As the molecular understanding of the biological functions necessary for metastasis increases, it becomes important to develop a comprehensive cancer metastasis chemoprevention strategy that targets not only the intrinsic growth of disseminated CTCs, but also their necessary adhesion and invasion to endothelial layer in the distant metastatic organs, including the bone marrow. Most of CTCs die in the circulation, presumably due to the loss of matrix-derived survival signals, circulatory shear stress, or anoikis [16]. Mifepristone, aspirin, lysine and doxycycline hyclate work differently at distinct metastatic targets and pathways.

Mifepristone is a progesterone receptor antagonist that has been widely used as the abortifacient and in anti-cancer trials [29]. We have comprehensively updated the information about the clinical trials of mifepristone [29]. Mifepristone prevented or delayed mammary tumorigenesis in the Brca1/p53-deficient mice [30], produced cancer cellular apoptosis by acting on p53 and B-cell lymphocyte/leukemia-2 (Bcl-2) family proteins [31], and induced a significant time- and dose-dependent cytotoxicity in both human androgen-sensitive LNCaP and androgen-insensitive PC3 and DU145 prostate cancer cell lines [32]. The inhibition of mifepristone is associated with a significant increase in DNA fragmentation, down-regulation of Bcl-2, and induction of TGF-beta 1 protein. It has been shown that mifepristone inhibited the invasive and metastatic potential of tumor cells through inhibition of the heterotypic adhesion to basement membrane, cell migration and angiogenesis [33]. Our latest study showed that mifepristone prevented colorectal HT-29 and breast MDA-MB-231 cell lines from migration, and interfered with the adhesion of cancer cells to endothelial cells [17]. Besides, mifepristone significantly decreased expression of focal adhesion kinase that is related to cell spreading and survival. Interestingly, it seems like that there are some similarity between embryo implantation and tumor metastasis [34], which constructs the base for the abortifacient mifepristone to act as a metastatic chemopreventive. Patients already took mifepristone for as long as 14 years [35]. The safety profile of mifepristone makes it well-suited for a safe metastatic chemopreventive candidate.

Aspirin is a widely-used anti-inflammatory drug, which inhibit COX-2 [also known as prostaglandin endoperoxide synthase 2 (PTGS2, or HGNC9605)]. Use of aspirin that inhibits platelet activation in the colon cancer cases is associated with improved overall survival [36]. Aspirin is a potential adjuvant therapy to prevent distant metastasis in colorectal cancer, and possible other cancers [37]. The targets on which the beneficial effects of aspirin are exerted have been identified in many cancer types. Pharmacologic data on aspirin indicate that systemic concentrations of aspirin, reached with low doses (75-325 mg once daily), are inadequate to permanently acetylate COX-2 but are optimal for its metastatic chemoprevention through inhibition of platelet activation and thrombocytosis [38], inhibition of MMP-2 activity and increase in E-cadherin production [39], down-regulation of an epithelial-mesenchymal-like phenotype [40], and inhibition of platelet-mediated nuclear factor-kB signaling in CTCs. We demonstrated that HAMPT exerted an inhibitory effect on ADP-induced platelet activation that could cloak CTCs from host immune attack and facilitate adhesion of CTCs to endothelium for consequent metastasis. Collectively, aspirin in HAMPT could inhibit adhesion and invasion of CTCs to the vascular endothelium and their intravascular micrometastasis formation. Regular aspirin intake has been shown to reduce risk of distant metastasis by 30-40% and reduce risk of metastatic adenocarcinoma by almost half [41-42]. We, therefore, propose that aspirin may be a good metastasis chemopreventive ingredient of HAMPT.

Doxycycline hyclate has been safely used as antibiotics effectively for decades [43]. It also exhibits antitumor activity in some tumor models and has potential for preventing bone metastasis and inhibiting cancer cell proliferation [44-45]. Doxycycline is also a potent MMP inhibitor and highly osteotropic [46-47]. Doxycycline has been shown to play an important role in reduction or prevention of cancer bone metastasis in preclinical and clinical settings [48].

Lysine is an essential amino acid and regarded as a non-bicarbonate/non-volatile buffer with a higher pKa at 10. Lysine inhibits MMPs, strengthens connective tissue surrounding cancer cells (tumor encapsulating effect), and reverses the acidity surrounding the CTCs microenvironment [49-50]. Animals treated with lysine for six weeks lived significantly longer than animals on tap water [51]. We considered lysine in HAMPT as a “soil-strengthening” agent that buffers the acidic extracellular pH, decreases proteolytic enzyme activity, and increases the ECM integrity. All of these could collectively lead to inhibition of CTCs extravasations and colonization.

Based on the evidence available in the literature and our own research [17], as well as the present study, we hypothesized that a combination of mifepristone, aspirin, lysine and doxycycline hyclate could work at a much lower but safe and effective concentration to prevent human cancer metastasis. The quantitative analysis of various combinations concludes that the quadruple combination HAMPT reached the maximum inhibition on tumor metastasis to lungs (Figure 4A, 4B).

**Figure 6:**
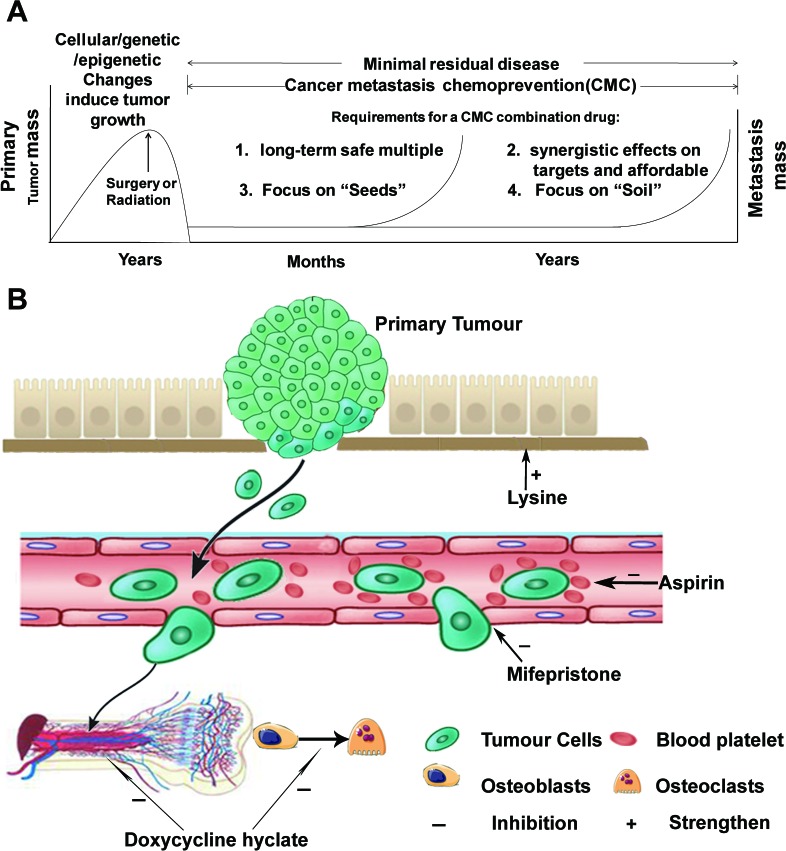
Cancer metastasis chemoprevention strategies **A.** The genetic, epigenetic, and cellular changes induce tumor growth that can be surgically removed. The residual disease may be detectable for long periods before the tumor seeds in distant secondary organs. To systematically prevent and restrain cancer metastasis, a true cancer metastasis chemoprevention combination drug should be safe, affordable, and synergistically target multiple metastatic pathways including both “seeds” and “soil”. **B.** the mechanisms of actions of HAMPT ingredients. Mifepristone produces cancer cell apoptosis by acting on various apoptosis-related targets; it inhibits adhesion and invasion of CTCs to vascular stroma by acting at both CTCs and endothelial cells as well as focal adhesion kinase. Aspirin inhibits COX-2 resulting in inhibition of platelet activation and thrombocytosis, and inhibition of cloak effect by platelet on CTCs. Doxycycline may inhibit the change from osteoblasts to osteoclasts, and interfere with bone marrow microenvironment to interrupt the reservoir for disseminating carcinoma. Both aspirin and doxycycline have the anti-inflammatory effect that is important for cancer metastasis prevention. Lysine strengthens extracellular matrix (soil) and buffers the acidic microenvironment.

On the basis of the findings from this study, we propose a consolidated model that illustrates a plausible sequence of events that orchestrate the mechanisms of actions of HAMPT in preventing and inhibiting cancer metastasis (Figure 6A, 6B). This model elucidates the potential role of HAMPT in controlling cancer metastasis cascade by acting on both the seeds and soil, and highlights the importance of using the safe and affordable drugs for long-term cancer metastasis chemoprevention after surgical removal of a primary tumor.

Old drugs may be “repurposed” for new indications and studied to determine the mechanisms of their beneficial and adverse effects [52]. A rational combination of those old and hence well-demonstrated effective and safe old drugs would be invaluable for cancer metastatic chemoprevention because the safety is the number one concern for those asymptomatic cancer survivors. The use of the affordable, safe, and efficient HAMPT that can be manufactured in a large quantity could theoretically and practically provide a rational basis for cancer metastasis chemoprevention, an important area that has been ignored for too long.

## MATERIALS AND METHODS

### HAMPT composition

Stock solution of HAMPT is composed of the following: aspirin 75 mg (Aladdin Reagent Company, China), lysine 200 mg (Sinopharm Chemical Reagent Co., China), mifepristone 10 mg (Shanghai New Hualian Co., China), and doxycycline hyclate 50 mg (Shanghai Civi Chemical Technology Co., China). Drug doses were chosen based on the human doses of the four drugs [36, 51, 53-54] and converted to mouse doses according to the body surface area index [24]. The concentrations of the four drugs used in the *in vitro* assay were chosen based on the same dose ratio of the four drugs composed and used in the animal studies. The combination was so formulated to target different pathways of cancer metastasis.

### Drug-drug interaction analysis by HPLC and UPLC-MS/MS

To determine whether physicochemical drug-drug interaction exists among the four drugs, we developed and validated the HPLC and UPLC-MS/MS methods similar to what we reported previously [17]. Briefly, the individual drugs were mixed in methanol-water 5:1 (v/v) at the same concentration as a single drug, i.e., 10 μg/mL. The HPLC system was composed of Waters 2695 pump equipped with Waters 2475 UV detector. Chromatographic separation was conducted on an analytical column (5 μm, 4.6×5 mm, Sigma Amide) using the mobile phase consisting of 0.1% formic acid in ultrapure water-methanol (30:70, v/v) at a flow rate of 1 mL/min. The injection volume was 20 μL and detection wavelength was at 280 nm.

UPLC-MS/MS analysis was carried out on a Waters H-class liquid chromatograph interfaced with XEVO-TQD Mass Spectrometer (Waters) equipped with heated capillary interface, electrospray ionization (ESI) source, and a tandem quadrupole mass detector. The ESI system employed a 3-kv spray voltage (positive polarity) and a heated capillary at 340°C. The auxiliary gas (nitrogen) flow rate was set to 1 L/min, cone voltage 27 V. The optimized collision energy was 25 V for lysine. The ESI was optimized using lysine as a reference compound. The mass chromatograms were acquired in total ion current (TIC) modality in MRM mode of the transitions of m/z 146.9→84.6 for lysine. Chromatographic analysis was the same as the HPLC condition except that the injection volume was 5 μL and the flow rate was 0.2 mL/min.

### Cell culture

The cell lines we used in our experiments were authenticated. The cell culture method were shown in supplementary materials.

### Hetero-adhesion of cancer cells to the endothelial cells and matrigel

Quantification of cancer cell adhesion to endothelial cells and Matrigel^TM^ (BD) was performed as we described previously with minor modifications [55]. Briefly, the human melanoma cells M619 transfected with the green fluorescent protein (GFP) were co-cultured with the human umbilical vascular endothelial cells (HUVECs) pretreated with IL-1β (1 ng/mL) for 4 h in the 24-well tissue culture plates. The human melanoma M619 and mouse melanoma B16-F10 cells were also cultured separately on the 96-well plates coated with Matrigel. The cell lines were treated with different concentrations of HAMPT for 1 h. The wells were washed with PBS, and the fluorescence signal of the M619-GFP cells attached to the HUVECs was recorded using a fluorescence microscope (Zeiss) to determine the effect of HAMPT on cell-cell adhesion, or the MTT assay was applied to determine the effect of HAMPT on adhesion of both human and mouse melanoma cells to Matrigel by using our method [17].

### Cancer cells cloaked by platelets

Blood was collected from healthy volunteers who had not taken any anticoagulants for at least 14 days prior to the study. Blood (1.8 mL) was added to the blood collection tubes containing 0.2 mL of anticoagulator sodium citrate (106 mM), and gently invert-mixed. Cancer cells were harvested after incubated with 0.25% trypsin solution and resuspended in PBS (2×10^6^ cells/mL). The cloak effect of platelets on cancer cells was measured by the flow cytometry. Briefly, HAMPT (0, 1, 10 and 30 μg/mL) and M619 cells (2×10^5^ cells) were added to 100 μL blood samples in falcon tubes followed by addition of ADP (20 μM) to stimulate the platelet aggregation. After 10 min incubation at 25°C, 20 μL mouse anti-human CD61 (FITC-labeled) and 20 μL mouse anti-human epithelial cell adhesion molecule (EpCAM, PE-labeled) were added to the tubes, respectively. The mixture was incubated for 20 min at 25°C in the dark followed by addition of 1 mL ice-cold 1% paraformaldehyde for fixture. After 30 min incubation of the samples at 4°C, cancer cells cloaked by platelets were determined by fluorescence-activated cell sorting (FACS), and only the CD61^+^EpCAM^+^ cancer cells were considered platelet-cloaked cancer cells. The data were processed by FlowJo software and expressed as the number of CD61^+^EpCAM^+^ cells.

### Cell viability and cell cycle analyses

The viability assay was similar to what we described previously [56]. Briefly, the melanoma B16-F10 and M619 cells were cultured as usual in 96-well plates at 8×10^3^ cells/well, and incubated with HAMPT (0-100 μg/mL) for 24 h at 37°C before the 4-h MTT assay. The result was read at OD_570nm_ on an ELISA reader. The untreated control was considered 100% viability.

The cell cycle was analyzed as we described before [57]. Briefly, M619 and B16-F10 cells were separately treated with different concentrations of HAMPT (0, 10, 30 and 50 μg/mL) for 24 h. The cells were harvested, washed twice with PBS and fixed in 70% ice-cold ethanol overnight, and then spun to remove ethanol before cellular DNA staining with the fluorescent solution (1% (v/v) Triton X-100, 0.01% RNase, 0.05% PI) for 30 min at 37°C in darkness. The cell cycle distribution was determined by flow cytometry.

### Cell adhesion molecules analysis by flow cytometry

This experiment was conducted as we described previously [16]. Briefly, the well-grown HUVECs were pretreated with IL-1β (1 ng/mL) for 4 h followed by HAMPT treatment for 4 h. The cells were collected and incubated at 4°C for 30 min in the dark with the primary antibodies. B16-F10 cells were treated with HAMPT for 24 h without IL-1β followed by incubation with anti-mouse CD49d-FITC antibodies (1:50 dilution) for 30 min at 4°C. Expression of cell-surface ICAM-1, VCAM-1 and α_4_ integrin (fibronectin receptor) was measured by the FACS AriaIII flow cytometer. The data were processed by FlowJo software and expressed as the mean fluorescent intensities.

### *In vivo* metastasis assay

All the drugs in this experiment were dissolved in 0.5% Carboxy Methyl Cellulose-Na (CMC-Na, purchased from Sinoharm Chemical Reagent Co., China) as a suspension. The mice were randomly divided into 4 groups (*n* = 6 per group), including CMC-Na (control) and HAMPT (33.5, 67 and 134 mg/kg/day) groups. The chemopreventive HAMPT was given by gavage for 4 days before B16-F10 inoculation followed by 30-day treatment. The immune-intact C57BL/6 mice were inoculated with the minimal metastatic B16-F10 cells (3×10^4^/0.2 mL/mice). Thirty days after the inoculation, the mice were sacrificed, and their lungs were excised. The number of surface melanoma colonies was counted visually with the aid of a dissecting microscope. The livers were also dissected, fixed in 10% (v/v) buffered formaldehyde. Sections of the lungs were stained with hematoxylin-eosin (H&E) to confirm that the nodules were malignant and to monitor the presence of micro-metastases foci. Sections of livers were used to determine whether HAMPT caused toxicity to livers. Overall survival of the mice treated with or without HAMPT were estimated using Kaplan-Meier survival curves as described by Xie et al [58].

Survival times were calculated from the date of inoculation. Mice that died before the end of the experiment were examined at the time of death to analyze the reasons of death. Cox proportional hazards and logistic regression models were fitted to identify the factors significantly associated with the survival rates.

### Synergistic or additive combination test *in vivo*

To examine the synergistic or additive effect among the four drugs, dual and triple combinations were made among the four drugs, and their effects were compared with that of HAMPT *in vivo*. The dose chosen for the test was the median dose of each individual drug in HAMPT (mifepristone 2 mg/kg/day, aspirin 15 mg/kg/day, lysine 40 mg/kg/day and doxycycline hyclate 10 mg/kg/day) and the experiment was conducted the same as described above in the *in vivo* metastasis assay.

### Acute and sub-acute toxicity assay

The assay was similar to what we described previously [25-26]. The assay was exerted by using KM mice and SD rats, and the details were shown in supplementary materials.

### Statistical analysis

The data were presented as mean ± SD of three determinations. Statistical analysis was performed using the Student's *t*-test and one-way analysis of variance. Multiple comparisons between the means were done by the least significance difference (LSD) test. P values less than 0.05 were considered statistically significant. All computations were made by employing SPSS statistical software (version 19.0).

## SUPPLEMENTARY MATERIAL TABLE


