# Supplementary material for: Aspirin, lysine, mifepristone and doxycycline combined can effectively and safely prevent and treat cancer metastasis: prevent seeds from gemmating on soil
Source: Oncotarget. 2015 Oct 8;6(34):35157–72. doi: 10.18632/oncotarget.6038 (PMC4742096; doi:10.18632/oncotarget.6038)
Supplement: Supplementary file 1 [file oncotarget-06-35157-s001.pdf]

Aspirin, lysine, mifepristone and doxycycline combined can effectively and safely prevent and treat cancer metastasis: prevent seeds from gemmating on soil

Supplementary Material

Materials and methods

Cell culture

The cell lines we used in our experiments were authenticated. The cell culture method was similar to what we described before [1]. Briefly, human melanoma cancer cells M619 and mouse melanoma cells B16-F10 were cultured separately in the RPMI 1640 medium supplemented with 10% fetal bovine serum, penicillin (100 U/mL), and streptomycin (100 μg/mL) in tissue culture flasks. Cells were incubated in 4 mL of media at 37°C in a tissue culture incubator equilibrated with 95% air and 5% CO_2. The human umbilical vascular endothelial cells (HUVECs) were prepared in our laboratory as described previously [2] and grew in the M199 medium supplemented with 20% FCS, 50 mL endothelial cell growth factor, heparin (5 U/mL), and antibiotics. The third passage of the HUVECs was used for the study.

Monoclonal antibody

Anti-mouse CD49d (α_4 integrin) labelled with fluorescein isothiocyanate (FITC) was obtained from eBioscience. The mouse anti-human CD61-FITC (integrin αv) was obtained from Becton Dickinson (BD) Pharmingen^{TM}, CD106 (VCAM-1), CD54 (ICAM-1) and CD326 (Epithelial cell adhesion and activating molecule, EpCAM) were all labelled with P-phycoerythrin (PE), and also obtained from BD Pharmingen^{TM}.

Animals used in in vivo metastasis assay

C57BL/6 mice (20 ± 2 g, 6-8 weeks old) were purchased from Shanghai SLAC Laboratory. Mice were maintained with free access to pellet food and water in microinsulator cages on a 12-h light/12-h dark schedule for 1 week. All animals used in the investigation were handled in accordance with the Guide for the Care and Use of Laboratory Animals (National Research Council, 1996), and approved by the institutional animal care and use committee of Fuzhou University. All possible efforts were made to minimize the animals' suffering and reduce the number of animals used.

Acute and subacute experiments

For the acute toxicity study, male and female adult Chinese Kun Ming (KM) mice with an average weight of 20 g were divided into four groups of 20 each (10 males and 10 females). The animals were separated by gender and housed 10 in each cage under the same conditions as mentioned above for the mice, and the assay was exerted for two weeks. For the subacute toxicity study, Sprague-Dawley (SD) rats (females with an average weight of 140 g and males with an average weight of 180 g) were randomly divided into four groups of 10 each (5 males and 5 females). The experiment began after a 1-week acclimation period and conducted similar as described previously for both acute and subacute experiment [3].

References

1. Shao J, Dai Y, Zhao W, Xie J, Xue J, Ye J, Jia L. Intracellular distribution and mechanisms of actions of photosensitizer Zinc(II)-phthalocyanine solubilized in Cremophor EL against human hepatocellular carcinoma HepG2 cells. Cancer Lett. 2013; 330: 49-56.
2. Lu Y, Yu T, Liang H, Wang J, Xie J, Shao J, Gao Y, Yu S, Chen S, Wang L, Jia L. Nitric oxide inhibits hetero-adhesion of cancer cells to endothelial cells: restraining circulating tumor cells from initiating metastatic cascade. Sci Rep. 2014; 4: 4344.
3. Jia L, Schweikart K, Tomaszewski J, Page J G, Noker P E, Buhrow S A, Reid JM, Ames MM, Munn DH. Toxicology and pharmacokinetics of 1-methyl-d-tryptophan: absence of toxicity due to saturating absorption. Food Chem Toxicol. 2008; 46: 203-211.

Table S1. Analysis of synergistic or additive effect of the different combinations among mifepristone (M), Aspirin (A), Lysine (L), Doxycycline hyclate (D)

Group	"q" value	Category	Group	"q" value	Category
M+A	1.63	Synergistic	L+D	1.12	Additive
M+L	1.45	Synergistic	L+(M+A)	1.06	Additive
M+D	1.07	Additive	L+(M+D)	1.19	Synergistic
M+(A+L)	1.20	Synergistic	L+(A+D)	1.13	Additive
M+(A+D)	1.15	Synergistic	L+(M+A+D)	1.11	Additive
M+(L+D)	1.12	Additive	D+(M+A)	1.01	Additive
M+(A+L+D)	1.08	Additive	D+(M+L)	1.03	Additive
A+L	1.12	Additive	D+(A+L)	1.13	Additive
A+D	1.12	Additive	D+(M+A+L)	1.08	Additive
A+(M+L)	1.06	Additive	(M+A)+(L+D)	1.08	Additive
A+(M+D)	1.21	Synergistic	(M+L)+(A+D)	1.09	Additive
A+(L+D)	1.11	Additive	(M+D)+(A+L)	1.18	Synergistic
A+(M+L+D)	1.10	Additive			

[a] i.e. The q value of A+(M+L) = $E_{A+(M+L)}/(E_A+E_{(M+L)}-E_A\times E_{(M+L)})$. Where, $E_{A+(M+L)}$, E_A, and $E_{(M+L)}$ are the average inhibitory effect of the triple combination (A+M+L), effect of drug A alone, and effect of dual drug combination (M+L), respectively. (M+L) means a dual combination drug of mifepristone and lysine.
